# Epigenetic Mechanisms Involved in HCV-Induced Hepatocellular Carcinoma (HCC)

**DOI:** 10.3389/fonc.2021.677926

**Published:** 2021-07-15

**Authors:** Pin Zhao, Samiullah Malik, Shaojun Xing

**Affiliations:** ^1^ Guandong Key Laboratory for Biomedical Measurements and Ultrasound Imaging, School of Biomedical Engineering, Shenzhen University Health Science Center, Shenzhen, China; ^2^ Department of Pathogen Biology, Shenzhen University Health Science Center, Shenzhen, China

**Keywords:** hepatitis C virus, hepatocellular carcinoma, DNA methylation, histone modifications, signaling pathways

## Abstract

Hepatocellular carcinoma (HCC), is the third leading cause of cancer-related deaths, which is largely caused by virus infection. About 80% of the virus-infected people develop a chronic infection that eventually leads to liver cirrhosis and hepatocellular carcinoma (HCC). With approximately 71 million HCV chronic infected patients worldwide, they still have a high risk of HCC in the near future. However, the mechanisms of carcinogenesis in chronic HCV infection have not been still fully understood, which involve a complex epigenetic regulation and cellular signaling pathways. Here, we summarize 18 specific gene targets and different signaling pathways involved in recent findings. With these epigenetic alterations requiring histone modifications and DNA hyper or hypo-methylation of these specific genes, the dysregulation of gene expression is also associated with different signaling pathways for the HCV life cycle and HCC. These findings provide a novel insight into a correlation between HCV infection and HCC tumorigenesis, as well as potentially preventable approaches. Hepatitis C virus (HCV) infection largely causes hepatocellular carcinoma (HCC) worldwide with 3 to 4 million newly infected cases diagnosed each year. It is urgent to explore its underlying molecular mechanisms for therapeutic treatment and biomarker discovery. However, the mechanisms of carcinogenesis in chronic HCV infection have not been still fully understood, which involve a complex epigenetic regulation and cellular signaling pathways. Here, we summarize 18 specific gene targets and different signaling pathways involved in recent findings. With these epigenetic alterations requiring histone modifications and DNA hyper or hypo-methylation of these specific genes, the dysregulation of gene expression is also associated with different signaling pathways for the HCV life cycle and HCC. These findings provide a novel insight into a correlation between HCV infection and HCC tumorigenesis, as well as potentially preventable approaches.

## Introduction

Hepatocellular carcinoma (HCC), is the third leading cause of cancer-related deaths with about 1 million deaths annually worldwide (1, 2). Around 70% to 90% of all patients with chronic liver diseases will develop hepatocellular carcinoma (3). By summing up the main risk factors worldwide, researchers have discovered that chronic hepatitis B virus (HBV) infection is the main reason in eastern Asia and sub-Saharan Africa, whereas in North America, Europe, and Japan, hepatitis C virus (HCV) infection and alcohol abuse are the main factors (2). Here we try to discuss the relationship between HCV infection and hepatocellular carcinoma in an epigenetic aspect.

Hepatitis C virus (HCV) is a single-stranded, positive-sense RNA with approximately 9,600 nucleotide bases. The RNA genome is initiated for translation and transcription in host cells (4). The studies on replication system of HCV life cycle are crucial for our understanding of acute and chronic HCV infection. There are several steps of HCV life cycle, including attachment, endocytosis, fusion, HCV RNA translation, proteolytic processing, viral RNA replication, assembly, maturation, and release (5). It begins with viral attachment to two host receptors, low-density lipoprotein receptor (LDLR) and heparin sulfate proteoglycans (HSPGs), which triggering HCV outer membrane protein E1/E2 heterodimer to bind to CD81 and scavenger receptor B1 (SRB1) (6–8). It is reported that initial attachment not only ensures a tight junction between HCV and hepatocytes, but also possibly leads to HCV particle degradation (9). HCV particle is then folded into an internal endosome by hepatocyte cell membrane and clathrin, as a result of interaction between CD81 and Claudin1 (CLDN1) (10, 11). Along with viral entry into the cell, clathrin is dissociated into the cytosol and then HCV RNA is also released into the cytosol through the acidic pH-dependent endosome fusion and endocytosis (12). Subsequently, HCV RNA, combined with ribosome in rough endoplasmic reticulum, functions as a template for HCV polyprotein translation. A single polyprotein precursor with approximately 3000 amino acids is translated and further processed into 10 mature viral proteins, including structural and nonstructural glycoproteins (E1, E2, p7, NS2, NS3, NS4A, NS4B, NS5A, and NS5B). RNA-dependent RNA polymerase, NA5B, generates a negative-sense RNA intermediate with the template of HCV RNA for the synthesis of numerous HCV RNA. HCV assembly, maturations, and release are complex. During assembly, HCV core proteins form a protective shell with HCV RNA. This immature HCV particle recruits a luminal lipid droplet (LuLD) to create a high-density HCV precursor. The pre-very-low-density lipoprotein (pre-VLDLs) is formed in rough endoplasmic reticulum (13). In the Golgi, the orderly combination of pre-VLDLs, large triacylglycerol (TG)-rich lipid droplets, and high-density HCV precursors is required for the cellular secretory machinery to transport these multivesicular bodies to the cell surface (14, 15).

Since HCV was discovered, it has been reported to be tightly associated with HCC. For example, HCV replication has been detected IN HCC tissue (16, 17). Overexpression of HCV core protein causes HCC in transgenic mice (18). And HCV infection in HepG2 (human hepatoblastoma) cells promotes cell growth and tumor formation *in vivo* (19). However, HCV-induced HCC is a long-term process that progresses more than 20 years and involves a serial of steps, including chronic HCV infection, chronic hepatic inflammation, liver fibrosis, steatosis, cirrhosis, irreversible genetic or epigenetic alterations, and progression of the malignant carcinogenic cells (20). Different from HBV-induced HCC by integrating its DNA into the host genome and leading to potential directly tumorigenesis, the carcinogenic potential of HCV is considered to indirectly link to HCV infection, inflammation, and genetic or epigenetic alterations. Some studies showed that HCV infection induced oxidative stress by viral proteins. Oxidative stress is essential for inflammatory progression and promote immunological tolerance in hepatitis. The genotype 3 of HCV is reported to more associated with steatosis and fibrosis progression in HCV patients (21).

As a member of the *Flaviviridae* family, hepatitis C virus (HCV) is a major risk factor of liver cirrhosis and hepatocellular carcinoma (HCC). With approximately 71 million HCV chronic infected patients worldwide, they still have a high risk of HCC in the near future, although direct-acting antivirals (DAA) powerfully eliminate most chronic HCV infection in patients (22). However, some multiple epidemiological studies demonstrate that after DAA treatment, sustained virologic responses (SVR) disturbs normal interferon regulation, which is helpful for HCC tumor development and metastasis (23–25). Moreover, liver transplantation and the transmission of undiagnosed chronic HCV infection will also exceptionally increase the risk of HCV infection. Some vaccines are designed to replace these direct-acting antivirals (DAA) by interrupting HCV transmission or stimulating the production of neutralizing antibodies (nAbs) (26). However, there is not yet an effective vaccine to prevent HCV infection. A number of challenges to cure HCV infection have aroused people to uncover more molecular mechanisms of the immune response to dysfunction.

One of the remarkable molecular alterations in HCV-infected patients is the dysregulation of some specific genes and signaling pathways, which resulted from dynamic epigenetic changes in the genome. Epigenetic regulation is specifically dictated by DNA methylation, post-translational histone modifications, chromatin remodeling, and noncoding RNA-mediated gene silencing. Previous results showed that HCV infection induces genome-wide epigenetic changes in active and repressed chromatin, where altered histone modifications and DNA methylation of the host gene decide their mRNA levels and expression. And then these changes influence the downstream signaling pathways associated with the HCV life cycle and HCC (27). Besides, infection with HCV generally leads to chromosomal instability by condensing chromatin and forming multi-centrosomes (28). In this review, we will focus on epigenetic changes induced by HCV-infected HCC tumorigenesis, including DNA methylation, histone modifications, non-coding RNAs, and related signaling pathways.

## Methylation Profiles in HCV-Infected HCC

**Figure 1:**
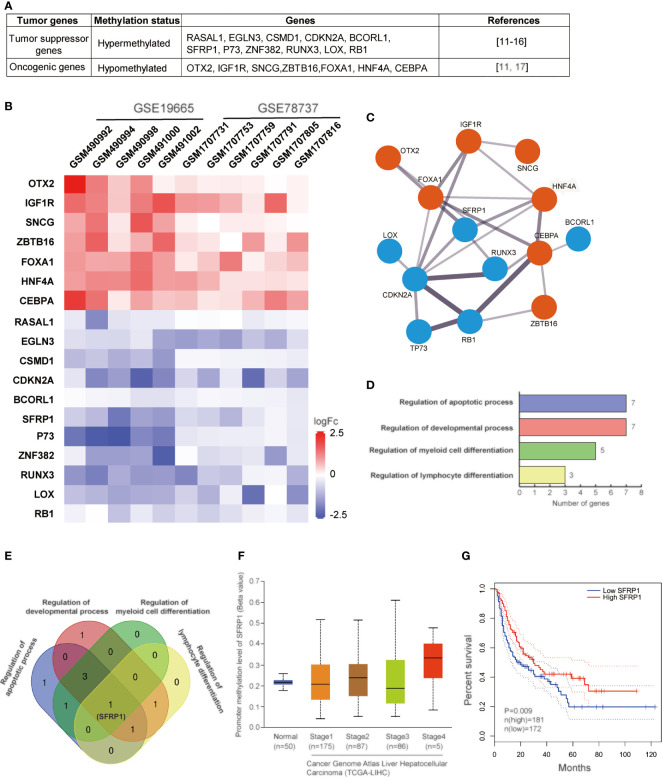
Methylation analysis of genes in HCV-induced HCC. **(A)** Table of 11 tumor suppressor genes and 7 oncogenic genes regulated in HCV-induced HCC. **(B)** Heatmap of the correlation between the expression levels of 18 genes and HCV-infected HCC samples among TCGA data sets. The output thresholds are FDR < 0.05, P < 0.05 and |log2 FC| > 1. **(C)** Functional interaction network of 14 genes. Protein interactions are presented by STING online tool (https://stingnetwork.com). Blue dots indicate down-regulated, red, up-regulated. Line thickness indicates the strength of interactions. **(D)** The biological processes with 18 genes are analyzed. There are four different processes involving in corresponding genes. Gene Ontology analysis is conducted using online website PANTHER Tools (http://pantherdb.org/data/). **(E)** Venn results of 18 genes which are regulated in the four biological processes. It is showed that only SFRP1 is commonly regulated in all biological processes. **(F)** The DNA methylation levels of SFRP1 in clinicopathological stages of HCC in the TCGA data set. **(G)** Kaplan–Meier curves showing that patients with different expression levels of SFRP1 had different overall survival.

DNA methylation-related alterations, including DNA hypermethylation, hypomethylation, and imprinting loss, tightly associated with various human diseases, like cancers. Specifically, DNA hypermethylation at the promoter regions of some tumor suppressor genes represses the expression of these genes, which immediately contributes to the carcinogenic processes of HCV-infected HCC. And hypermethylation of promoter CpG island is the main mechanism of these genes’ inactivation. Studies have shown that the methylation disorders for the inactivation of tumor suppressor genes and activation of oncogenic genes are the one common reason for the occurrence of liver cancer. However, our understanding of liver-specific genes remains quite limited. In summary, 18 genes, including tumor suppressors and oncogenic genes, are methylated with different levels (29–35) (**Figure 1A**). RNA-seq analysis has shown that promoter hypermethylation of tumor-suppressive genes contributes to low protein and hypomethylation of oncogenic genes leads to relatively high expression (**Figure 1B**). To investigate the properties of the gene regulatory network in HCV-induced HCC, we mapped these 18 genes by STING network. It showed that there were 14 genes involved in and SFRP1 displayed most connected nodes and edges (**Figure 1C**). Also, these genes are involved in different cellular processes (**Figure 1D**). It is shown that cell apoptotic and development processes are the most influenced in HCC cells with the greatest number of enriched genes, and cell differentiation is the second, which suggests that HCV infection induces HCC mainly through dysregulation of cell apoptosis and development.

Except for tumor suppressor, p53, a number of tumor suppressor genes have also been reported to be hypermethylated in previous researches. There are 11 tumor suppressor genes involved in HCV-infected HCC development, including RASAL1, EGLN3, CSMD1, CDKN2A, BCORL1, SFRP1, ZNF382, RUNX3, LOX, RB1, P73. In general, hypermethylation of these genes transcriptionally represses protein expression, thus all of these tumor suppressor genes are downregulated in HCV-related HCC cells.

RAS protein activator like 1 (RASAL1) is a member of the GTPase-activating proteins (GAPs) family. By transforming the GTPase activity of Ras proteins into the inactive GDP-bound form of Ras, RASAL1 inhibits Ras/MAPK signaling. The hypermethylation of RASAL1 leads to low protein expression, which stimulates cell proliferation and differentiation through Ras/MAPK signaling in HCV-induced tumorigenesis. RASAL1 has been consistently reported to be downregulated in HCC cells (36).

Egl-9 family hypoxia inducible factor 3 (EGLN3) is one of the hypoxia-related prognostic genes and correlated with a poor prognosis in HCC (37). The family of prolyl hydroxylases (PHDs/EGLNs) is reported to inhibit tumor growth through degradation of hypoxia-inducible factor (HIF) (38). Therefore, HCV infection may downregulate EGLN3 expression, which stimulates HCC development.

CUB and Sushi multiple domains 1 (CSMD1) is a membrane-bound complement inhibitor, of which lncRNA, lncCSMD1, is associated with tumor progression of HCC cohorts (39). The expression of lncCSMD1 elevates MYC protein in the nucleus of HCC cells, resulting in cell proliferation and invasion. The activation of the MYC signaling pathway promotes tumor growth and metastasis of HCC patients. In addition, miR-10b binds to the 3′-untranslated region (3’-UTR) of CSMD1 for downregulating CSMD1 expression, whereas miR-10b is highly expressed in HCC tissues compared to normal tissues (40).

CDKN2A, also known as cyclin-dependent kinase inhibitor 2A by binding to cyclin-dependent kinases (CDKs), acts as a tumor suppressor by blocking the cell cycle from G1 to S phase (41). Besides P53, CDKN2A is also mostly inactivated in human cancers. It is first discovered that DNA hypermethylation occurs at the CDKN2A promoter region in the plasma and serum of liver cancer patients (42). And subsequently, CDKN2A promoter methylation is reported to increase HCC risk in tissues (43). Meta-analysis has validated that promoter methylation of CDKN2A links to an enhancive HCC risk and likely to be a triage marker for HCC (44). Deletion or knockdown of CDKN2A in HCV-induced HCC tissues is also common, as well as mutations of TP53, AXIN1, and CDKN2A (45).

BCL6 corepressor like 1 (BCORL1) is abnormally expressed in human HCC (46). It is reported that BCORL1 prominently promotes facilitated epithelial-mesenchymal transition (EMT) of HCC by negatively regulating E-cadherin expression. BCORL1 protein is a transcriptional corepressor and can interact with several histone deacetylases to repress the transcription of E-cadherin. It is suggested to be an independent prognostic marker for human hepatocellular carcinoma.

Zinc-finger protein 382 (ZNF382), as a member of the zinc-finger protein family, has been evidenced to be downregulated in several types of cancers. In hepatitis B virus (HBV)-related HCC cells, ZNF382 is commonly downregulated by promoter hypermethylation (47). Tumor suppressor ZNF382 mainly impairs of Wnt/β-catenin pathway and activates p53 signaling to induce cell apoptosis in HCC cells.

RUNX family transcription factor 3 (RUNX3) is reported to act as a tumor suppressor in breast cancer and lung cancer. It is known to transcriptionally regulate genes associated with the development and differentiation of cells within the immune and nervous system. In HCC cells, promoter hypermethylation of RUNX3 is reported to occur at an early stage of HCC development (48). Knockdown of RUNX3 stimulates expression of a multidrug resistance-associated protein (MRP) and results in chemotherapy resistance in HCC (49). Consistently, RUNX3 restoration downregulates cancer stem cell (CSC) signaling in hepatocellular carcinoma through Jagged1-Notch signaling (50).

Lysyl oxidase (LOX) is a copper‐dependent amine oxidase for cross‐linking of collagen and elastin molecules in the extracellular matrix. In HCC cells, LOX induces epithelial–mesenchymal transition (51). And the expression of LOX is reported to be regulated by HIF‐1α or hypoxia. However, it is still unclear about the connection between the expression of LOX and early recurrence and poor survival in patients with HCC.

RB transcriptional corepressor 1 (RB1) is a key regulator of multiple cellular processes, such as stability of chromatin structure, DNA replication, and cell cycle. As a negative regulator of the cell cycle, RB1 functions to control cells from dividing too fast. Only when it is phosphorylated by CDK3/cyclinC, RB1 promotes G0/G1 transition in the cell cycle. RB1 exerts its tumor suppression by interacting with E2F1, subsequently repressing its transcription activity (52, 53). Therefore, RB1 is frequently reported to be downregulated in many types of human cancers. It is evident that RB1 genes are silenced in HCC, but different from other cancers, RB1 is rare to be mutated in HCC cells (54). Moreover, low expression of RB1 in HCC cells mainly contributes to promoter methylation (55).

Tumor protein P73 is a homology of p53 protein with a similar DNA binding domain and frequency of mutation in cancers. In contrast to p53, p73 is poorly understood. It is reported that p73 expression is significantly related to HCC prognosis and could be an indicator of HCC patients (56). However, this study concludes that P73 is upregulated in HCC cells, which is controversial to other research results. It has been reported that the promoter methylation level of P73 is significantly higher in HCC than in normal tissues (57). So, it is still necessary to clarify the expression level and methylation level of P73 in HCC patients.

One of the important genes we mentioned above is SFRP1. Secreted frizzled-related protein 1 (SFRP1) functions as a regulator of the Wnt signaling pathway by interacting with Wnts. By inhibition of Wnt signaling, SFRP1 expression increases, and thus, it is classified as a tumor suppressor. In various human cancers, SFRP1 is transcriptionally silenced *via* DNA methylation (58). The high expression level of SFRP1 has a meaningful impact on survival rates of HCC patients, even though the underline mechanisms are still unknown. HCV infection promotes epigenetic inactivation of SFRP1, which is helpful for hepatocellular carcinoma. It has been evident that promoter methylation of SFRP1 upregulated in all stages of HCC and low SFRP1 expression decreases HCC patient’s survival rate (**Figures 1E–G**). Similar to another solid tumor, the development of HCC is thought to require at least three dysregulations of cellular processes (proliferation, cell cycle, apoptosis) (59). Epigenetic repression of SFRP1 may cause dysregulation of cell proliferation, cell cycle, migration, and differentiation, which result in cancer cell formation and poor prognosis, and drug-treated resistance. Hence, SFRP1 protein expression provides the possibility for a new therapy approach of HCV-induced HCC.

As for oncogenic genes, it mainly includes ZBTB16, OTX2, IGF1R, SNCG, FOXA1, HNF4A, CEBPA. The high expression of Zinc Finger and BTB Domain Containing 16 (ZBTB16) and orthodenticle homeobox 2 (OTX2) have also been found. However, their functions in HCC remain to be further studied. Type 1 receptor (IGF1R) is reported to interact with insulin-like growth factor 1 and 2 (IGF1 and IGF2) for increasing fibrosis and impair liver functions from the start of preneoplastic alterations up to the developed hepatocellular carcinoma (HCC) stage (60). The neuronal protein, synuclein-gamma (SNCG), is highly expressed in advanced hepatocellular carcinomas, and SNCG gene activation *via* demethylation in tumor tissue of HCC is not limited to HBV and HCV infection (35). Collectively, both IGF1R and SNCG is regarded as a potential indicator that demethylation of their CpG islands is an early sign of genetic abnormality in liver cirrhosis preceding hepatocarcinogenesis.

Forkhead box A1 (FOXA1) is a transcription factor and involved in embryonic development, metabolism, and epithelial lineage differentiation (61). It is reported that the expression of FOXA1 is relatively higher in both HCC tissues and human hepatic cell lines than non-tumor tissue and cells. It is concluded that FOXA1 promotes cell proliferation and inhibits apoptosis (62), whereas FOXA1 is also found to suppress hepatocellular carcinoma cell viability and motility by transcriptional inactivation of phosphoinositide-3-kinase regulatory subunit 1 (PIK3R1) in PI3K/Akt signaling (63). Therefore, the functions of FOXA1 as an oncogenic gene or tumor suppressor are still controversial. The mechanism of FOXA1 in the development of HCC is necessary to be further explored.

Hepatocyte nuclear factor 4a (HNF4a) is a member of liver-enriched transcriptional factors and regulates hepatic gluconeogenic program and lipid metabolism by binding to specific genomic regions of particular genes (64). It is reported that HNF4a expression is associated with aggressive HCC (65). Recent evidence has explained that two isoforms of HNF4a are determined by two separated promoters, P1 and P2. P1-HNF4a is distributed in normal adult liver, whereas P2 products are in fetal liver and liver cancer (66). The reduced nuclear P1-HNF4a contributes to proliferation and inflammation with HCC risk (67). In contrast, P2-HNF4a is induced in HCC (68). P2-HNF4a prevents P1-HNF4α re-localization from the nucleus to cytoplasm for tumor growth by downregulating the circadian clock gene ARNTL in healthy hepatocytes. However, to date, the role of HNF4a in HCC progression is still to be elucidated.

CCAAT/enhancer-binding protein alpha (CEBPA) is also a transcriptional factor, which contains a basic leucine zipper (bZIP) domain and recognizes the CCAAT motif in the promoter of target genes. CEBPA is reported to inhibit cell proliferation, cell motility, and metastasis. And thus, CEBPA is known as a putative tumor suppressor, and it is downregulated in solid tumors (69). In the liver, it also functions in liver homeostasis and hematopoietic myeloid cell lineage. By activating CEBPA in the HCC tumor microenvironment with a first-in-class small activating RNA oligonucleotide drug, MTL-CEBPA, white cell count significantly increases, and HCC progression delays in combination with Sorafenib treatment (70). Functional alterations of CEBPA have also been investigated in HCC cell lines. It is upregulated in a subset of HCC (71). In HCC tissues, CEBPA expression epigenetically promotes cell proliferation, which suggests a novel oncogenic mechanism in HCC.

HCV-infected HCC has been reported to associate with some repetitive elements (REs) in non-coding regions of the genome, mainly including long interspersed nuclear element-1 (LINE1) and Alu element (Alu). It is reported that DNA methylation of this locus-specific REs is down-regulated in HCV-induced HCC, although the molecular mechanisms in liver cancer development are still unclear (72). In addition, gene silencing induced by DNA methylation is governed by DNA methyltransferases (DNMTs). The expression level of DNMTs is also influenced by HCV infection. It has been reported that different HCV genotypes regulate both mRNA and protein expression of DNMTs. For example, mRNA levels of DNMT1, 3a, 3b are upregulated in genotype 1b and 3a, and DNMT 3b mRNA is also increased in genotype 2a (73).

## Histone Modifications in HCV-Infected HCC

Histone modifications have been intensively studied in various cancer models, especially histone methylation, acetylation, phosphorylation, sumoylation, and ubiquitination. During HCC development, histone methylation, and acetylation are widely investigated. It has been reported that putative vasohibin 2 (VASH2) abnormally overexpresses to promote HCC proliferation and inhibits apoptosis, which resulted from the lower H3K27 trimethylation, and higher high H3K4 trimethylation and H3 acetylation at VASH2 promoters (74).

For the roles of histone methylation on special HCV-induced HCC, it has been reported that overexpression of KDM5B/JARID1B, a member of JmjC histone demethylase, could enhance HCC cell proliferation through the regulation of its downstream genes, E2F1 and E2F2 (75). Another histone lysine-specific demethylase (LSD1) is also upregulated, and its expression was linked with HCV infection in HCC. With the Kaplan-Meier method, LSD1 protein is shown to decrease rates of overall survival and disease-free survival and regarded as an independent prognostic factor in HCC patients. LSD1 knockout cells display a higher level of H3K4me1/2 and H3K9me1/2 than normal cells (76). Histone lysine or arginine methylase, including G9a, EZH2, SUV39H1, and SUV39H2, tightly associate with the progression of HCC prognosis. G9a, a histone methyltransferase for lysine 9 of histone 3 (H3K9), mainly functions in gene inactivation, which is essential for early embryogenesis (77, 78). A synthetic triazole analog of guanosine for anti-HCV therapy, ribavirin (RBV) exerts its function by downregulating the mRNA levels of several preactivated interferon (IFN)-stimulated genes (ISGs) in HCC patients. By recruiting more G9a, these selected ISGs are modified with histone H3 lysine9 dimethylation/trimethylation (H3K9me2/H3K9me3) for transcription repression. Defect of G9a impairs ISG downregulation and inhibits RBV to activate IFN for anti-HCV action (79). EZH2 is a histone methyltransferase for lysine 27 of histone 3, of which gene belongs to polycomb. EZH2 plays a crucial role in gene silencing, chromatin remodeling through the interaction with other transcription factors (80). The overexpression of EZH2 is regarded as a promising biomarker for HCC patients in China (81). To patients with HCV infection to HCC, there are not only DNA methylation of targeted enhancers, enriched for the binding sites of the transcription factors, FOXA1, FOXA2, and HNF4A, and decreased histone H3 lysine 27 trimethylation (H3K27me3), caused by the EZH2 inhibitor GSK343, but also negatively associated with increased CG dinucleotide density and polycomb-mediated repression on genes involved in stem cell development (34). SET domain-containing histone lysine methyltransferase 1 (SUV39H1) is also upregulated in HCC patients from Hong Kong, but not patients from the Japanese population (82, 83). However, compared with SUV39H1, SUV39H2 is more correlated with HCV infection (84).

Histone acetylation, regulated dynamically by histone acetyltransferases (HATs) and histone deacetylases (HDACs), also widely occurs in the development of HCV-infected HCC, such as histone H3 acetylated on lysine 9 (H3K9Ac), histone 3 acetylated on lysine 27 (H3K27Ac), H2A acetylated on lysine 5 (H2AK5ac), and H3 acetylated on lysine 14 (H3K14Ac) (85, 86). Knocking out histone deacetylase 3 (HDAC3) immediately leads to hyperacetylated H3K9, along with lower trimethylation of H3K9 (H3K9me3) in mice, which disturbs double-strand break (DSB) repair and promotes HCC tumorigenesis (87). H3K27Ac, mainly localized at central euchromatin regions in cancer cells, activates global cell type-specific gene expression. HCC tissues also show higher H3K27Ac levels than the normal liver no matter it is caused by HCV infection or HBV infection (88). HCC-related histone acetylation usually involves multiple biological processes. For example, iron overload is a risk factor of HCV to HCC progression in the liver, which is normally regulated by a small peptide, human hepcidin, produced by our body. With HCV infection, the expression level of hepcidin is decreased by histone deacetylase (HDAC) activation on histone acetylation, not DNA methylation (89). The role of hepcidin against HCV replication with signal transducer and activator of transcription 3 (STAT3) signaling activation provides a potential treatment for anti-HCV defense. Furthermore, histone deacetylase (HDAC) has been reported to be positively associated with HCV and HCC. The inhibitor of HDAC, therefore, has been considered for clinical HCC treatment because people found that HDAC3 inhibitor suppressed HCV replication by increasing liver-expressed antimicrobial peptide 1(LEAP1) and decreasing apolipoprotein A1 (ApoA1). The antiviral effect of HDAC3 inhibitor may be explained to alter histone acetylation of these gene promoter regions with the transcription factors, including hypoxia-inducible factor 1α (HIF1α) and STAT3 (90).

Measurement of dynamic changes on histone modifications in specific HCV-induced HCC remains limited for characterizing clinical features and treatments of HCC. With the development of detection methods, including immunohistochemistry, Western blot, and ChIP-sequencing, more specific alterations on histone status and DNA–protein modifications will be traced for potential epigenetic therapies in HCV-specific HCC.

## Regulation of Noncoding RNAs (ncRNAs) in HCV-Infected HCC

High throughput transcriptome studies have discovered abundant noncoding RNAs in eukaryotes. These novel RNAs seem to involve in a larger scope of cellular functions beyond our understanding. Noncoding RNAs (ncRNAs) play a critical role in the regulation of various cancers, including HCC (91). In these 18 selected genes, several genes are tightly associated with ncRNAs. For example, the promoter region of SFRP1 is reported to be methylated by non-coding RNA HOTTIP-recruited DNMT3B and promotes inflammation in rheumatoid arthritis (92). And lncRNA-maternally expressed gene 3 (lncRNA-MEG3) upregulates IGF1R expression in hypoxia-induced proliferation of pulmonary artery smooth muscle cells (PASMCs) proliferation (93). FOXA1 expression is activated by long noncoding RNA (lncRNA) SBF2-AS1 and repressed by lncRNA PVT1 (94, 95). lncRNA CEBPA-AS1 promotes tumorigenesis *via* CEBPA/Bcl2 in oral squamous cell carcinoma (96). However, the mechanisms by which ncRNAs regulate HCV-induced HCC need to be further explored. Two kinds of ncRNAs, including microRNAs (miRNAs) and long noncoding RNAs (lncRNAs), have been most studied in recent years.

MicroRNAs (miRNAs) are small non-coding RNA with 20-23 nucleotides in length and originated from 60 to 70 nucleotides miRNA precursor (pre-miRNA), which play a crucial regulatory role in various cellular functions by targeting mRNAs for mRNA degradation and translational repression (97–99). Aberrant miRNAs profiles have been displayed in human cancers, including HCC. In HCV infection HCC cells, miRNAs exert an antiviral effect on HCV replication and infection through virus–host interaction (100). More than 1,000 miRNAs have been identified to date. Among them, miR-122 is the one of most abundant miRNAs in the liver and tightly associated with HCV replication and HCC (101, 102). It is reported that miR-122 binds 5′-UTR of the HCV genome and promotes the replication and translation of HCV RNA (103). Moreover, the upregulation of miRNA-122 in human HCC cells results from the transcription activation of the DR1 and DR2 motifs at its promoter by binding peroxisome proliferator-activated receptor-gamma (PPARγ) and retinoid X receptor alpha (RXRα) complex (104). However, hepatitis B virus X protein (HBX), not HCV protein, binds PPARγ and suppresses miR-122 gene transcription. Viral infectivity was enhanced by miR-122 but significantly reduced by miR-200c. miR-200c is reported to target the 3′UTR of occludin (OCLN) and reduce OCLN mRNA level, and therefore, prevents HCV entry to cells (105). HCV infection also decreases miR-124 level *via* Dnmt1, resulting in cell migration and invasion in intrahepatic cholangiocarcinoma (ICC) cells (106). In addition, miR-182 is dysregulated in HCV-infected NK cells, which influences HCV replication (107). Also, miRNA-484, 524, 615, and 628 are also discovered in HCV-mediated HCC patients and are potential important biomarkers for diagnosis of fibrosis and cirrhosis (108).

LncRNAs are non-coding RNAs that are more than 200 nucleotides in length, which participate in various biological processes and associate with human disease. Current evidence points to lncRNAs affect the diverse cellular functions of the immune response, including innate and adaptive immunity (109, 110). IFN is an antiviral factor to prevent viral entry, replication, transcription, translation, packaging, and release. Recent studies have shown that several lncRNAs positively or negatively regulate the IFN response (111). For example, lncRNA 8 (Lnc-ITM2C-1/LOC151484) has been identified to be increased for HCV replication in HCV-infected Huh-7.5 hepatoma cells (112). It positively regulates HCV infection by indirectly promoting the expression of G protein-coupled receptor 55 (GPR55) and repressing the expression of several interferon-stimulated genes (ISGs), including ISG15, Mx1, and IFITM1 during the IFN response. In contrast, lncRNAs are regulated by interferon-alpha (IFNα) in Huh7.5.1 cells, of which gRNA significantly inhibits HCV infection through its spatial domain. And also, IFI6 is negatively regulated by lncRNAs (113). As for HCV-related HCC, there are only two lncRNAs reported, including HIF and PAR5 (114). These lncRNAs are significantly dysregulated in HCV-related HCC tissues. Other seven lncRNAs, including LINC01419, BC014579, AK021443, RP11-401P9.4, RP11-304 L19.5, CTB-167B5.2, and AF070632, are differently expressed and could be potential biomarkers for the early detection of HCC (115).

## Signaling Pathways in HCV-Induced HCC

**Figure 2:**
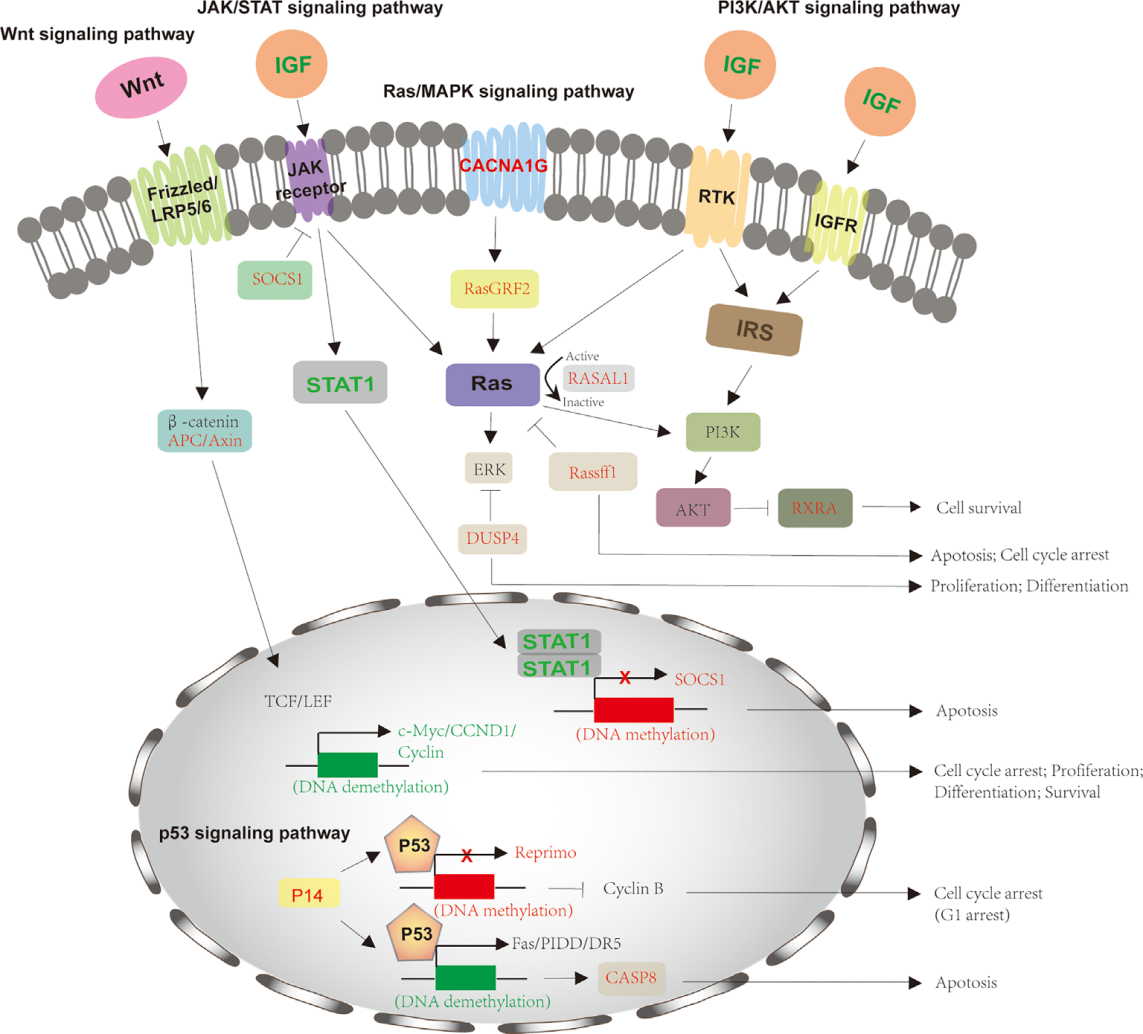
Signaling pathways involved in HCV-induced HCC. The process of HCV-induced HCC involves in several signaling pathways, including Wnt signaling pathway, Ras/MAPK signaling pathway, p53 signaling pathway, JAK-STAT pathway, and PI3K-AKT pathway. For Wnt signaling pathway, HCV infection downregulates APC and Axin2 expression *via* hypermethylation, which activates LEF/TCF-dependent downstream genes, such as c-Myc, CCND1, and Cyclin and thus promotes cell proliferation and differentiation. The HCV-mediated activation of JAK-STAT pathway by decreasing SOCS1 and increasing IGF, STAT1 to forms a negative feedback and inhibits cell apoptosis. The downregulation of DUSP4, RASAL1, and RasGRF2 by HCV infection also stimulates Ras/MAPK signaling pathway for cell growth. IGF also activates PI3K-AKT pathway by reducing RXRA expression. p53 signaling pathway is also modulated by HCV infection through downregulation of P14, Reprimo and CASP8, preventing from cell cycle arrest and apoptosis. The crosstalk between different signaling pathways happens and cooperates for cell growth and tumorigenesis. The proteins with normal expression are black; the downregulated proteins, resulted from these gene promoters’ DNA methylation, are red and upregulated proteins for gene promoters’ DNA demethylation are green. APC, adenomatous polyposis coli; DUSP1, dual‐specificity phosphatase 1; JAK/STAT, Janus kinase-signal transducer and activator of transcription; IGF, insulin-like growth factor; CCND1, cyclin D1; SOCS1, suppressor of cytokine signaling 1; RASAL1, Ras protein activator like 1; RasGRF2, Ras protein-specific guanine nucleotide-releasing factor 2; CASP8, caspase 8; MAPK, mitogen-activated protein kinase.

The molecular mechanisms of HCV-induced HCC are complex. Recent evidence has shown that HCV RNA-encoded proteins can modulate several signaling pathways, including the Wnt signaling pathway, Ras/MAPK signaling pathway, p53 signaling pathway, JAK-STAT pathway, and PI3K-AKT pathway (**Figure 2**). These pathways are significantly linked with cell cycle regulation, proliferation, and apoptosis. The dysregulation of signaling pathways by HCV proteins is obvious in the development of HCC.

## Wnt Signaling Pathway

The conserved Wnt signaling pathway regulates numerous cellular processes, including cell polarity, motility, migration, and neural patterning during embryonic development (116). More than 50% of HCC patients display an alteration in the Wnt signaling pathway, including protein expression and mutation (117). And around 30% of HCC occurs mutation of β-catenin for its accumulation (118). Wnt is a secreted, hydrophobic cysteine-rich lipid-modified glycoprotein (119). It is bound by cell surface receptor (Frizzled receptor) and co-receptor (low-density lipoprotein–related protein 5 or 6 [LRP5/6]) and activated Wnt to prevent the β-catenin degradation complex from degradation (120). This complex is composed of casein kinase I, glycogen synthase kinase 3β (GSK3β), adenomatous polyposis coli (APC), Diversin, and Axin, which phosphorylate β-catenin and subsequently induce β-catenin degradation by the proteasome (121). The downregulation of APC or Axin2 by HCV infection, to some extent, leads to the accumulation of de-phospho-β-catenin, thereby more β-catenins translocate to the nucleus for the aberrant activation of LEF/TCF target genes, such as c-Myc, CCND1, and Cyclin. Furthermore, β-catenin accumulation also stimulates the malignant transformation of hepatocytes to HCC cells by overexpressing Deltanp73 for ant-apoptosis and chemoresistance of cells (122, 123). Deltanp73 is a repressor of tumor suppressor p53 and its human homology tap73 (124). In addition, when HCV causes chronic liver injuries, including liver fibrosis, epigenetic downregulation of peroxisome proliferator-activated receptor γ (PPARγ) is the main reason for myofibroblastic transdifferentiation (MTD) during liver fibrogenesis, which is proved to be associated with the Wnt signaling pathway. Inhibition of the Wnt signaling pathway restores PPARγ expression and hepatic stellate cells (HSCs) differentiation for liver regeneration (125). These studies suggest that the Wnt signaling pathway notably regulates HCV-infected HCC.

## Ras/MAPK Signaling Pathway

The aberrant activation of the Ras/MAPK pathway has been evident in the occurrence and development of HCC (126, 127). This pathway is activated by various extracellular growth factors-mediated cell surface receptors, either receptor tyrosine kinase (RTK) or G‐protein‐coupled receptor (GPCR). Ras, a small GTPase, plays a key role in this pathway. Once activated by these growth factors, GTP-Ras recruits Raf1 to phosphorylate MAP2Ks and ERK 1/2, activating two key transcription factors of the AP‐1 family, c‐Jun and c‐Fos for expression of downstream genes involved in cell cycle and differentiation, through the translocation of ERK into the nucleus (128, 129). In this pathway, RASSF1, a member of the Ras association domain family (RASSF), is a Ras inhibitor, of which the expression is reduced by the DNA methylation of its gene’s promoter in the condition of HCV infection (130). The member of GTPase‐activating proteins (GAPs), RASAL1, promotes the switch of GTP to GDP, causing Ras from GTP-activated form to GDP-inactivated form (131). However, it is downregulated by DNA methylation during the development of HCV-induced HCC. And dual‐specificity phosphatase 1(DUSP1) is a specific ERK regulator (132). It is also downregulated by HCV infection. For the HCV replicon system, activation of the MEK1/2 kinase activity of Ras/MAPK signaling pathway inhibits the anti-HCV effect of IFN-gamma, which is partly because of the direct or indirect regulation of NS5A protein phosphorylation (133). The Ras/MAPK signaling pathway has a key role in HCV infection and the development of HCC.

## PI3K/AKT Signaling Pathway

PI3KAkt/PKB plays a major role in cancer development and progression by inhibiting apoptosis and stimulating cell proliferation. This pathway is activated by multiple growth factors (IGF and EGF) and cytokines (134). Once activated, phosphoinositol-3-kinase (PI3K) then activates Akt, a serine-threonine kinase, through a lipid messenger phosphoinositol triphosphate 3 (PIP3). By phosphorylating multiple downstream substrates from cytoplasmic proteins, Akt regulates various cellular activities, such as cell proliferation, cell division, apoptosis, and RNA processing. It has been reported that PI3K/Akt signaling is activated by HCV, which promotes HCV entry and replication (135, 136). Akt phosphorylation occurs when PI3K activates SREBP, a downstream effector of this signaling pathway, resulting in HCV translocation by HCV NS5A. NS5A can protect cells from apoptosis, which is one underlying mechanism that has been uncovered. Specifically, the tumor suppressor gene, PTEN, is a negative regulator of the PI3K/Akt pathway by dephosphorylating PIP3 and inactivating Akt. Downregulating phosphatase and tensin homolog (PTEN) by NS5A enhances PI3K/Akt pathway and accumulates more Akt for HCV-infected HCC pathogenesis (137). In addition, myeloid-derived suppressor cells (MDSCs) function to maintain HCV persistent infection, and MDSC-like monocytes are induced by HCV through PI3K/Akt signaling pathway (138). Taken together, the PI3K/AKT signaling pathway strongly associates with the life cycle of HCV, and its inhibition could be a promising HCC treatment strategy.

## JAK/STAT Signaling Pathway

Janus kinase-signal transducer and activator of transcription (JAK/STAT) regulates multiple cellular functions, such as differentiation, proliferation, and apoptosis, by transmitting extracellular signals to the nucleus for the transcriptional activation or repression of genes. It has been reported that this signaling pathway plays a crucial role in HCV-induced HCC. Especially, when stimulated, HCV-infected cells with interleukin 6 (IL6), the phosphorylation of JAK1/2 and STAT3, and STAT3-mediated transcription are inhibited by HCV core-binding JAKs, whereas IFN-gamma stimulation promotes JAK1/2 phosphorylation and STAT3-mediated transcription (139). The mechanistic analysis shows that HCV prevents the effect of IFN stimulation on JAK/STAT signaling pathway by degrading components in this pathway (140). Furthermore, the downregulation of STAT promotes virus replication and prevents anti-viral IFN-stimulated genes (ISG) expression, including PKR, OAS2, MxB, and ISG15 (141).

The expression level of signaling-related protein STAT1 is significantly down-regulated in the HCC tumor tissues, of which the underlying mechanisms are complex (142). On one hand, although STAT1 is essential for HCV replication, it is obviously decreased in HCV-infected cells as a result of proteasome-dependent degradation by HCV core/E1/E2, and NS3-4A proteins (143). On the other hand, STAT1 inhibits HCC growth by promoting p53-related cell cycling and apoptotic cell death in the STAT1 signaling pathway (142). Also, a recent study uncovers that STAT1 overexpression induces G0/G1 cell cycle arrest *via* downregulated expressions of cyclin A, cyclin D1, cyclin E, and CDK2 protein and promotes apoptosis by decreasing the expression of NF-κB p65 and Hes-1 and increasing the expression of p53 and Fbxw7 (144).

In addition, activation of JAK-STAT signaling and IFN-stimulated genes (ISG) expression is also regulated by a novel innate immune regulator, stromal antigen 2 (encoded by STAG2) (145). STAG2, a subunit of nuclear cohesion complex, plays an important role in genomic DNA stability, activation of cGAS-STING-IRF3 signaling, and recognition of cytoplasmic microchromatin. The deficiency of STAG2 in cells inhibits virus replication *via* the activation of JAK-STAT signaling.

## P53 Signaling Pathway

P53 tumor suppressor is a nuclear transcription factor that regulates several genes involved in apoptosis, cell cycle arrest, and growth. It is activated by genotoxic or cellular stresses like oxidative stress, DNA damage, and nutrient deprivation. In normal tissues, p53 restricts tumor growth and malignant transformation, not only by autonomously triggering cell cycle arrest or apoptosis but also by non-autonomously releasing senescence-related secretory phenotype in the tumor microenvironment (146). However, the P53 signaling pathway is one of the main apoptosis pathways. As a tumor repressor, p53 negatively regulates the progression of various cancers in some ways, one of which is called the MDM2-p53 feedback pathway (147). The alterations of the murine double minute 2 (MDM2) -p53 feedback pathway are common in HCC (148–150). MDM2 is an E3 ubiquitin ligase. Specifically, P53 binds the promoter region of MDM2 and activates MDM2 transcription, whereas the expressed MDM2 blocks P53 transcription through the interaction of the P53 transactivation domain and promotes P53 proteasome-dependent degradation. Mutations of p53 in HCC mainly occur in the DNA-binding domain of p53, which weakens the affinity of p53 to its target genes and reduces MDM2 expression. Mutations of P53 occur at an early stage of tumor development and fail to induce p53-mediated apoptosis or sentence, which disturbs cancer therapy. Therefore, mutations of the *P53* gene lead to patients with poorer survival rates for reduced expression of normal p53 protein.

Recent evidence has shown that p53 is overexpressed in the early stage of HCV-induced HCC, especially at HCV-infected damaged liver (151). HCV core-protein is reported to enhance p53 transcriptional activity and repress p21(waf1) expression. By acetylating *lys273* and *lys382* and phosphorylating *ser15*, HCV core-protein augments the ability of p53 to bind promoter regions of downstream genes (152). P21 inhibits cyclin-dependent kinase (CDK), and the HCV-mediated downregulation of p21 promotes cell cycle progression and cell growth. However, it is also reported that HCV nonstructural protein 5A (NS5A), transcriptionally represses p53 transactivation by directly interacting with p53 (153). Also, a coactivator of p53, hTAFII32, also binds to HCV NS5A, which incorporately inhibits p53-mediated transcriptional activation during HCV infection and may promote hepatocarcinogenesis. When p53 is knocked down in Huh7 cells, HCV RNA replication and viral protein significantly increase, suggesting that the p53 signaling pathway negatively regulates HCV infection and subsequently tumor development (154).

## Conclusions

HCV infection is a major etiological factor for liver diseases. About 80% of the infected people develop a chronic infection, which eventually leads to a more severe form of liver disease, including liver cirrhosis and hepatocellular carcinoma (HCC). Over 90% of primary liver cancer belongs to HCC, and it may progress through dysregulation of a complex epigenetic network (155). The alterations in transcriptional regulation, along with downstream signaling pathways, might play a critical role in HCV-induced HCC. In this review, we have summarized 18 genes (7 genes with hypomethylation and 11 genes with hypermethylation) that are commonly regulated for HCV-mediated hepatocarcinogenesis. Perhaps, there are a wide variety of genes, including these 18 genes, which are transcriptionally activated or repressed through site-specific DNA methylation at genes’ promoter regions. Complementing our upstream regulations is the activation or blocking of tumor-associated signaling pathways, indicating that HCV infection plays a crucial role in the development of HCC.

HCV infection induces the alterations of genomic methylation levels in the promoter regions of cellular genes, which transcriptionally influence protein expression. These dysregulations of proteins directly or indirectly promote tumor growth through various signaling pathways. In detail, the inactivation of cellular genes by DNA hypermethylation, especially tumor suppress genes promotes a chronic HCV infection to liver cirrhosis, even HCC. And demethylation of signaling-related genes and oncogenic genes is also an important cause of hepatic carcinogenesis resulting from HCV infection. For application, DNA methylation of cancer-related genes might be useful as a prognostic marker. However, detection of candidate gene methylation was started with HCV-infected HCC patients, and further study is necessary to explore the functional involvement in the development of hepatic carcinogenesis.

Aberrant regulation of histone modifications is also linked to hepatocellular carcinogenesis. On one hand, histone enzymes, including various acetyltransferases and demethylase, are directly influenced by HCV infection and tumorigenesis. On another hand, the dynamics of histone modifications on different targeted genes, especially histone acetylation for gene activation and methylation for gene silencing, happens in the process of HCV infection and HCC development. Further studies on histone modifications are helpful for exploring clinical biomarkers or potential treatment on hepatic carcinogenesis. Moreover, small RNAs are believed to regulate more than 60% of whole human genes (156). Accumulated evidence suggests that small RNAs are tightly involved in cancer. The aberrant expressions of microRNAs (miRNAs) and small non-coding RNAs influence the levels of various protein expressions, which could play key roles in cell proliferation and differentiation and HCC patients’ survival rate in the condition of HCV infection.

As mentioned above, no one pathway acts alone but signaling cross-talking between diverse pathways exists in liver cancer. The interplay between pathways becomes complicated and influents positively or negatively. For example, the upstream protein Ras is involved in various pathways, such as MEK/ERK, PI3K/AKT, and RalEGF/Ral pathways. And in turn, activating Raf and Akt gene simultaneously Ras/MEK and PI3K/Akt pathways (48). The receptors of the JAK/STAT signaling pathway also stimulates the Ras/MEK pathway *via* Ras protein, as well as the receptors of the PI3K/Akt pathway. So, the alterations of DNA methylation, histone modifications, and ncRNAs, epigenetically impact downstream signaling pathways when HCV infection occurs. Taken together, epigenetic regulation of HCV-induced HCC is complex and necessary to be deeply studied. The clinical application of its epigenetic mechanisms is, therefore, being further evaluated.

Taken together, with our understanding of the molecular mechanism of HCV-indued HCC *via* a complex epigenetic network, different strategies that targeted these 18 proteins and five signaling pathways should be potentially investigated. In consideration that HCV-induced HCC transcriptionally lead to the activation of oncogenic genes and repression of tumor suppressor genes, a combination therapy may be necessary for targeting different proteins. Also, it is also a great strategy to identify some chemical drugs, which inhibit simultaneously more than two major signaling pathways. Besides, targeted drug delivery systems (DDS) can also be helpful to deliver these therapeutic drugs to specific tumor sites for HCV-induced HCC treatment (157).

## Author Contributions

PZ prepared the original draft. SM discussed and contributed to the manuscript writing. SX supervised the project and contributed to the final version of the manuscript. All authors have made a direct and intellectual contribution to the work. All authors contributed to the article and approved the submitted version.

## Funding

This work has been supported by the Shenzhen Science and Technology Program (KQTD20190929172538530) and the National Natural Science Foundation (81971492).

## Conflict of Interest

The authors declare that the research was conducted in the absence of any commercial or financial relationships that could be construed as a potential conflict of interest.

## Glossary

**Table:**

APC	Adenomatous polyposis coli
ApoA1	Apolipoprotein A1
BCORL1	BCL6 corepressor like 1
Bzip	Basic leucine zipper
CDKN2A	Cyclin-dependent kinase inhibitor 2A
CEBPA	CCAAT enhancer binding protein alpha
CSC	Cancer stem cell
CSMD1	CUB and Sushi multiple domains 1
DAA	Direct acting antivirals
DNMTs	DNA methyltransferases
DUSP1	Dual&dash;specificity phosphatase 1
EGLN3	Egl-9 family hypoxia inducible factor 3
EMT	Epithelial-mesenchymal transition
FOXA1	Forkhead box protein A1
GSK3&beta;	Glycogen synthase kinase 3&beta;
GPCR	G&dash;protein&dash;coupled receptor
GAPs	GTPase-activating proteins
HCV	Hepatitis C virus
HCC	Hepatocellular carcinoma
HSCs	Hepatic stellate cells
HNF4A	Hepatocyte nuclear factor 4-alpha
HATs	Histone acetyltransferases
H2AK5ac	H2A acetylated on lysine 5
H3K9Ac	Histone H3 acetylated on lysine 9
H3K14Ac	H3 acetylated on lysine 14
H3K27Ac	Histone 3 acetylated on lysine 27
H3K27me3	Histone H3 lysine 27 trimethylation
HDACs	Histone deacetylases
HIF	Hypoxia inducible factor
IGF1R	Insulin-like growth factor 1 receptor
ICC	Intrahepatic cholangiocarcinoma
IFN	Interferon
ISGs	Interferon (IFN) -stimulated genes
IL6	Interleukin 6
JAK/STAT	Janus kinase-signal transducer and activator of transcription
LEAP1	Liver-expressed antimicrobial peptide 1
LINE-1	Long interspersed nuclear element-1
LOX	Lysyl oxidase
LSD1	Lysine-specific demethylase
MDM2	Murine double minute 2
MRP	Multidrug resistance-associated protein
MDSCs	Myeloid derived suppressor cells
MTD	Myofibroblastic transdifferentiation
ncRNAs	Noncoding RNAs
NS5A	Nonstructural protein 5A
OCLN	Occludin
OTX2	Orthodenticle homeobox 2
P73	Tumor protein p73
PIK3R1	Phosphoinositide-3-kinase regulatory subunit 1
PIP3	Phosphoinositol triphosphate 3
PPAR&gamma;	Peroxisome proliferator activated receptor-gamma
PTEN	Phosphatase and tensin homologue
PHDs	Prolyl hydroxylases
RASAL1	RAS protein activator-like 1
RB1	RNA binding motif protein 1
RBV	Ribavirin
RTK	Receptor tyrosine kinase
RXR&alpha;	Retinoid X receptor alpha
RUNX3	RUNX family transcription factor 3
SFRP1	Secreted frizzled related protein 1
SNCG	Synuclein gamma
STAT3	Signal transducer and activator of transcription 3
SUV39H1	SET domain-containing histone lysine methyltransferase 1
SVR	Sustained virologic responses
VASH2	Putative vasohibin 2
ZBTB16	Zinc finger and BTB domain-containing protein 16
ZNF382	Zinc finger protein 382
